# Phylogeography of Recently Emerged DENV-2 in Southern Viet Nam

**DOI:** 10.1371/journal.pntd.0000766

**Published:** 2010-07-27

**Authors:** Maia A. Rabaa, Vu Thi Ty Hang, Bridget Wills, Jeremy Farrar, Cameron P. Simmons, Edward C. Holmes

**Affiliations:** 1 Center for Infectious Disease Dynamics, Department of Biology, The Pennsylvania State University, University Park, Pennsylvania, United States of America; 2 Oxford University Clinical Research Unit, Hospital for Tropical Diseases, Ho Chi Minh City, Viet Nam; 3 Fogarty International Center, National Institutes of Health, Bethesda, Maryland, United States of America; Duke University-National University of Singapore, Singapore

## Abstract

Revealing the dispersal of dengue viruses (DENV) in time and space is central to understanding their epidemiology. However, the processes that shape DENV transmission patterns at the scale of local populations are not well understood, particularly the impact of such factors as human population movement and urbanization. Herein, we investigated trends in the spatial dynamics of DENV-2 transmission in the highly endemic setting of southern Viet Nam. Through a phylogeographic analysis of 168 full-length DENV-2 genome sequences obtained from hospitalized dengue cases from 10 provinces in southern Viet Nam, we reveal substantial genetic diversity in both urban and rural areas, with multiple lineages identified in individual provinces within a single season, and indicative of frequent viral migration among communities. Focusing on the recently introduced Asian I genotype, we observed particularly high rates of viral exchange between adjacent geographic areas, and between Ho Chi Minh City, the primary urban center of this region, and populations across southern Viet Nam. Within Ho Chi Minh City, patterns of DENV movement appear consistent with a gravity model of virus dispersal, with viruses traveling across a gradient of population density. Overall, our analysis suggests that Ho Chi Minh City may act as a source population for the dispersal of DENV across southern Viet Nam, and provides further evidence that urban areas of Southeast Asia play a primary role in DENV transmission. However, these data also indicate that more rural areas are also capable of maintaining virus populations and hence fueling DENV evolution over multiple seasons.

## Author Summary

Dengue virus (DENV) is the cause of the most common vector-borne viral disease of humans, and is at particularly high prevalence in parts of Southeast Asia. Most studies of DENV transmission have focused on very local or international movement patterns, and have not explored how DENV moves through an endemic region. To address this issue, we employed newly developed phylogeographic methods to study patterns of spatial spread in 168 full-length DENV-2 genome sequences collected during a hospital-based study in southern Viet Nam, focusing on the Asian I genotype that recently emerged in this region. This analysis revealed that the urban population of Ho Chi Minh City plays a central role in the dispersal of the virus, and that DENV in this city tends to move along a gradient of population density. In addition, human movement between urban and rural areas was the most likely explanation for the rapid diffusion of DENV across southern Viet Nam following its introduction into Ho Chi Minh City. After reaching more rural areas, some virus lineages were maintained there for a number of years. These results therefore indicate that virological surveillance is necessary in both urban and rural populations.

## Introduction

Dengue viruses (DENV) are mosquito-borne RNA viruses (family *Flaviviridae*) that exist as four antigenically distinct viruses or serotypes (DENV-1 through DENV-4) and show complex immunological interactions within a human host and at the epidemiological scale [1]. Current estimates suggest that more than half of the world's population resides in dengue endemic areas, with 40 million symptomatic infections occurring annually, over two million of which may be severe enough to require hospitalization [2]. Although most DENV infections are asymptomatic, the virus is responsible for significant morbidity in the developing world, and places a considerable burden on health systems during periods of both endemic and epidemic transmission [3].

The burden of dengue is highest in Southeast Asia, where all four DEN viruses currently circulate. Viet Nam shows consistently high levels of DENV transmission, with the incidence of dengue hemorrhagic fever (DHF) and dengue shock syndrome (DSS) ranging from 36 to 405/100,000 population per year between 1998 and 2008 [4], [5], and the southern part of the country accounting for 85% of cases nationally [6]. Most of these cases occur in children, who experience an annual exposure risk of ∼10% [7], [8]. Hyperendemicity was observed in southern Viet Nam as early as the 1960s, with all four viruses discovered in mosquito specimens collected in and around Ho Chi Minh City (HCMC; formerly Saigon) just years after the first DHF epidemics swept through South Viet Nam and cities across Southeast Asia [9]. DHF epidemics were reported in children from rural villages on the Mekong River and in urban HCMC in 1963, although a suspected DHF outbreak occurred in the Mekong Delta region in 1960. The disease was first recognized in rural areas, and the movement of the virus was proposed to have followed human movement and commerce on the Mekong River [10], still a major transportation route between HCMC and the Deltaic provinces [11]. Although incidence tends to peak during the rainy season, the tropical monsoon climate and high human population densities in southern Viet Nam allow year-round transmission of DENV [8]. Rapid urbanization and socioeconomic changes in recent decades may have also contributed to the establishment of what is now a relatively stable, highly endemic transmission pattern in the region.

In recent years, a number of studies have utilized gene sequence data to investigate the spatial and temporal dynamics of DENV in a restricted geographic area [12], [13] or during a single epidemic period [14]. An understanding of the evolution and spatial spread of DENV in a larger, stably endemic region over a period of several years may provide important information on the origins of epidemic strains, reveal how and in what populations DENV are able to persist at low levels of infection when immunological and ecological conditions do not favor epidemic activity, and perhaps assist in the prediction of the transmission patterns of newly emergent DENV lineages.

To this end, the aim of this study was to reveal trends in the spatial dynamics of DENV-2 in southern Viet Nam by using full-length genome sequence data obtained from patients admitted to a tertiary referral hospital in HCMC. These data provide a unique opportunity to investigate the spatial dynamics of different lineages of DENV-2 within a geographical region characterized by high endemicity. By using recently developed phylogeographic methods we address the following key questions: (i) Does HCMC, as the primary urban center of the region, act as a source population for dengue viruses circulating in southern Viet Nam? (ii) Within HCMC, are the highest density population areas acting as foci of viral dispersal throughout the city and the region? (iii) At this scale, does DENV dispersal adhere to a predictable population- or density-based model of transmission?

## Materials and Methods

### Study population and data

DENV-2 genome sequences were obtained from dengue patients enrolled into a prospective clinical and virological study of dengue at the Hospital for Tropical Diseases in Ho Chi Minh City, Viet Nam. These data and the sampling, serotyping, virus isolation, and sequencing methods used have been described elsewhere [15]. Written informed consent was obtained from the patient or guardian prior to participation in the study, which was approved by the Hospital for Tropical Diseases and the Oxford University Tropical Research Ethical Committee. Along with demographic and clinical data, the date of sampling and geographic information (Longitude, Latitude) on the location of each patient's home were collected by research staff using a hand-held GPS device. A full list of sequences used, their GenBank accession numbers, and place of sampling are given in Table S1.

From 2001 to mid-2008, 187 full DENV-2 genome sequences were obtained from hospitalized dengue cases in southern Viet Nam, and their complete coding regions (10,176 nt) were manually aligned using Se-AL v2.0a11 (available from http://tree.bio.ed.ac.uk/software/). Of these, three were initially excluded from this analysis; two sequences were missing spatial or genetic data, and one sequence was obtained from a subject who identified his or her home as being far outside of the study area (Nghe An province, >950 km north of HCMC).

As a large hospital-based study that likely included only severe cases, we would not reliably expect to capture clustering at the finest spatial and temporal scales, including direct chains of transmission. To identify such fine-scale clustering, pairwise genetic distances between the 184 remaining sequences were determined using the HyPhy package, employing the HKY85 model of nucleotide substitution with global branch length estimation parameters [16]. Geographic distances (WGS84 ellipsoid) between patient homes were determined using longitude and latitude coordinates with the ‘sp’ package in R (version 1.2.9) [17], [18] (Figure S1). All sequences were categorized according to date of sampling, geographic distance and genetic distance, and potential short transmission chains were eliminated in order to prevent short-term focal transmission events from biasing the larger-scale spatial analyses. In total, 14 sequences were found to be close enough in date of sampling (<15 days) [19], [20], geographic distance (<0.8 km) [21]–[23], and genetic distance (<0.0001) to other viruses in the database that they may represent direct transmission events, and were thus excluded from analysis. The resulting data set of 170 full genome DENV-2 sequences, sampled between 2001 and mid-2008, consisted of 126 viruses of the Asian I genotype, 42 of the American/Asian genotype, and two of the Cosmopolitan genotype. Because of their small number, isolates of the Cosmopolitan genotype were assumed to be importations from outside of the study area and were removed from all analyses. The final 168 sequence data set included samples obtained from the majority of districts in HCMC (20/24 districts) and from nine additional provinces in southern Viet Nam – An Giang (AG), Binh Duong (BD), Binh Phuoc (BP), Dong Nai (DN), Dong Thap (DT), Long An (LA), Tay Ninh (TN), Tien Giang (TG), Vung Tau (VT) – covering an area of approximately 37,500 km^2^ and a population of nearly 20 million (∼532 persons/km^2^) [24].

### Phylogenetic analysis

Bayesian Maximum Clade Credibility (MCC) phylogenetic trees were inferred for the DENV-2 genome sequences of the American/Asian and Asian I genotypes separately using a Bayesian Markov Chain Monte Carlo (MCMC) method implemented in the BEAST package (v1.5.2) [25], which incorporates date of sampling information and returns rooted trees. A strict molecular clock, a GTR+Γ_4_ model of nucleotide substitution (determined by Modeltest v3.7; [26]) with three codon positions (substitution model, rate heterogeneity model, and base frequencies unlinked across all codon positions), and a Bayesian skyline coalescent model (five coalescent-interval groups) were used for all analyses, all of which have previously been shown to be appropriate for the analysis of DENV [15], [27], [28]. Very similar results (no major differences in topology or coalescent times) were obtained under a relaxed (uncorrelated lognormal) molecular clock model (results available from the authors on request). Three independent runs of at least 150 million generations were performed, with sampling every 10,000 generations, until all parameters had reached convergence with 10% removed as burn-in. This analysis allowed us to estimate times to the most recent common ancestor (TMRCA) for key nodes on the DENV-2 phylogeny of each genotype. Nodal support is expressed as Bayesian posterior probability values.

### Analysis of spatial structure and virus dispersal patterns

To assess the overall degree of spatial admixture and geographical structure among DENV-2 lineages in this region, we calculated values of the association index (AI) [29] and parsimony score (PS) statistics [30] for each genotype from the posterior samples of trees returned by BEAST using the BaTS program [31]. This method accounts for phylogenetic uncertainty in investigating phylogeny-trait correlations, with 1000 random permutations of tip locations to estimate a null distribution for each statistic. This program also allowed us to assess the level of clustering in individual locations using the monophyletic clade (MC) size statistic. The relationships among sequences were estimated on three spatial levels: (i) by province (10 spatial groups, 45 possible diffusion pathways), (ii) by population density within HCMC and by province (nine provinces and three regions within HCMC: ‘Superurban HCMC’ - population density >15,000/km^2^, ‘Urban HCMC’ - population density <15,000/km^2^ and >2500/km^2^, and ‘Suburban HCMC’ - population density <2500/km^2^; 66 possible diffusion pathways overall), and (iii) by geographic proximity and population density-based district groupings within HCMC and by province (11 regions within HCMC, nine provinces; 190 possible diffusion pathways).

The strength of support for viral exchange between individual locations at each of the spatial levels was inferred using a geographically-explicit Bayesian MCMC approach implemented in BEAST [32], with the same coalescent models and spatial groups as described above. This method estimates a reversible diffusion rate for each potential diffusion pathway among the predefined locations while simultaneously estimating evolutionary and coalescent parameters, thereby allowing quantification of the uncertainty in ancestral state reconstructions (i.e. ancestral geographic locations). Bayesian Stochastic Search Variable Selection (BSSVS) was used to identify the links between these locations among the posterior sets of trees that explain the most likely migration patterns among DENV-2 in southern Viet Nam. Bayes factor (BF) tests were used to determine the statistical significance of diffusion pathways among the geographic groups. To summarize the posterior distribution of ancestral location states, nodes in the MCC trees were annotated with the modal location state for each node using TreeAnnotator, and trees were visualized using FigTree (available at http://tree.bio.ed.ac.uk/software). To account for the potential effects of sampling bias, data from highly sampled geographic areas were randomly subsampled with replacement to create smaller data sets from each geographic location, and analyses were repeated 10 times for each subsampling scheme.

### Distance and gravity model-based spatial analysis

Because pathogen dispersal across geographic areas is often thought to be influenced by factors such as distance and human population density, we integrated both distance and population-based priors into the phylogeographic analysis to mimic the effects of these factors on the DENV population. To calculate the distances between the defined populations, centroids for relevant geographic regions were determined using R (version 2.9.1) [17] with the ‘sp’, ‘shapefiles’, and ‘maptools’ packages [18], [33], [34], and utilizing a map of Viet Nam (shapefile format) defining first and second level subnational administrative boundaries [35]. Distances (WGS84 ellipsoidal) between centroids were subsequently estimated [18]. Population data for each of the provinces of Viet Nam and the districts of HCMC in 2007 were obtained from the General Statistics Office of Viet Nam and the Statistical Office in Ho Chi Minh City, and were used as representative population information for all analysis [24], [36].

To obtain prior estimates corresponding to these distance and population-based parameters, simple gravity model calculations providing estimates of the movement of populations (and disease dispersal) (*C_ij_*) between community *i* (of size *P_i_*) and community *j* (of size *P_j_*) were calculated using the relation:
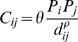
where θ is a proportionality constant and ρ adjusts the dependence of dispersal on the distance (*d*) between the two geographic areas [37]. Variables representing the dependence of dispersal on population sizes were not utilized due to the reversibility of the diffusion links assessed using this phylogeographic method, and could not have been reliably estimated due to a lack of data on differential population movements in the region. Normalized values (mean one and unit variance) for distance and gravity model calculations were then utilized as priors to inform the rates of diffusion among geographic locations. Diffusion rate prior distributions were fixed (F) or were sampled from multivariate Gamma prior (MGP) distributions. Distance and gravity model-based analyses were performed at the provincial level and at the second of the spatial levels (12 groups), but were not conducted using distance or population-informed priors at the finest spatial scale (HCMC district groups and provinces, 20 groups), as these regions were not comparable in terms of distances between centroids or population sizes.

## Results

### Overall patterns of geographic structure

Our initial trait association (AI and PS) tests of phylogeographic structure rejected the null hypothesis of no association between sampling location and phylogeny at all of the spatial levels tested for the Asian I DENV-2 genotype in southern Viet Nam (Table 1). Hence, these genome sequence data possess at least some geographic structure. The use of index ratios of the observed values to those expected under panmixis (in which 0 indicates complete population subdivision and 1 suggests random mixing [panmixis]) allows the strength of the association between geography and phylogeny to be characterized further. Accordingly, although panmixis was rejected by our analysis, the AI and PS index ratios for the Asian I genotype approached 1, indicating relatively frequent virus movement between geographic areas. In contrast, no significant associations between phylogeny and geography were observed at any of the spatial levels analyzed in the American/Asian genotype (Table 1), and BSSVS location reconstruction performed using American/Asian DENV-2 revealed no significant patterns of spatial diffusion, likely due to the small number of samples available (MCC trees shown in Figures S4 and S5). We therefore focused the rest of our study on the Asian I genotype.

**Table 1:** Phylogeny-trait association tests of phylogeographic structure of DENV-2 in southern Viet Nam.

Statistic	Spatial clustering	Index Ratio, observed to expected (95% CI)	Observed value (95% CI)	Expected value (95% CI)	P-value
**Association Index (AI)**					
Asian I	10 Provinces	0.60 (0.48–0.75)	5.3 (4.8–5.8)	8.9 (7.7–10)	<0.01
	9 Provinces, 3 HCMC	0.68 (0.59–0.81)	7.8 (7.4–8.3)	11.4 (10.3–12.5)	<0.01
	9 Provinces, 11 HCMC District Groups	0.69 (0.62–0.77)	9 (8.5–9.5)	13.1 (12.4–13.8)	<0.01
American/Asian	6 Provinces	0.82 (0.54–1.54)	1.4 (1.2–1.7)	1.7 (1.1–2.2)	0.18
	5 Provinces, 3 HCMC	0.92 (0.67–1.50)	2.3 (2–2.7)	2.5 (1.8–3)	0.31
	5 Provinces, 9 HCMC District Groups	0.84 (0.66–1.13)	3.1 (2.7–3.5)	3.7 (3.1–4.1)	0.06
**Parsimony Score (PS)**					
Asian I	10 Provinces	0.75 (0.72–0.80)	40 (40-40)	53.2 (50–55.8)	<0.01
	9 Provinces, 3 HCMC	0.84 (0.80–0.89)	64 (64-64)	76.3 (72.1–79.7)	<0.01
	9 Provinces, 11 HCMC District Groups	0.92 (0.89–0.97)	85.6 (85–87)	92.8 (89.6–95.9)	<0.01
American/Asian	6 Provinces	1.01 (0.90–1.00)	10 (10-10)	9.9 (9–10)	1
	5 Provinces, 3 HCMC	1.01 (0.94–1.00)	16 (16-16)	15.8 (15–16)	1
	5 Provinces, 9 HCMC District Groups	0.98 (0.91–1.09)	27.3 (27–28)	27.8 (25.8–29.7)	0.27
**Maximum clade (MC) scores** *					
Asian I	Dong Thap (DT)	NA	4 (4-4)	2 (1-3)	<0.01
	Tay Ninh (TN)	NA	2 (2-2)	1 (1-1)	<0.02
	Vung Tau (VT)	NA	2 (2-2)	1 (1-1)	<0.02
American/Asian	Ho Chi Minh City (HCMC)	NA	11.9 (11–16)	5.5 (3.2–9)	<0.03

*Only significant MC scores are indicated; for all other locations, p>0.05 as estimated using BaTS [31].

### Spatial analysis by province

**Figure 1:**
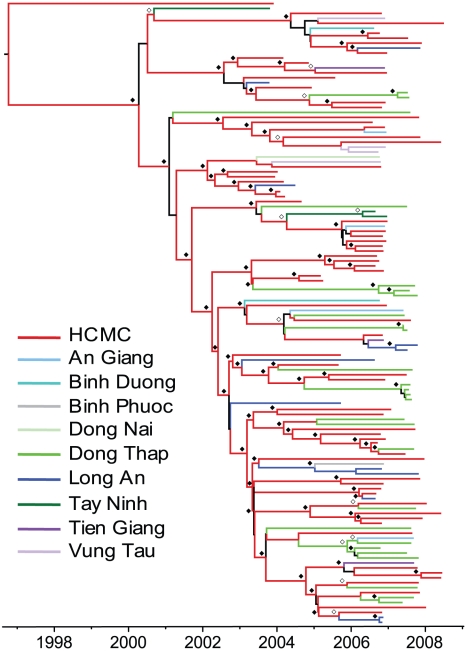
MCC phylogeny of the DENV-2 Asian I genotype in southern Viet Nam (2003–2008) according to province of sampling. Tips are colored by province of sampling. Internal branches are colored based on the reconstructed ancestral state as estimated by the reversible diffusion model. Branches colored black indicate Bayesian posterior probabilities less than 0.85. Estimated support for the reconstructed ancestral state is indicated by open (>95%) and closed (>85%) diamonds.

**Figure 2:**
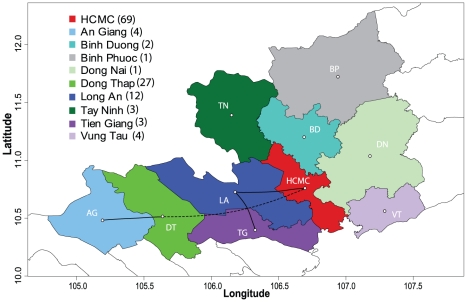
DENV-2 Asian I genotype dispersal across provinces of southern Viet Nam. Map showing significant pathways of diffusion estimated between provinces of southern Viet Nam. Solid lines indicate diffusion pathways among provinces with a shared border. Dashed lines indicate diffusion pathways among provinces that do not have a shared border. Numbers in parentheses indicate the number of sequences included in each group.

The MCC phylogeny of the Asian I genotype with BSSVS reconstructed ancestral locations (10 provinces) of the internal nodes reveals that viruses from nearly all provinces are dispersed throughout the phylogeny, most notably HCMC and DT, as well as LA and TG (Figure 1). Although some local clustering was observed, the general trend in the tree is of mixing among geographic locations, with Bayesian phylogeographic analysis estimating significant reversible diffusion pathways between AG and DT, DT and HCMC, HCMC and LA, and LA and TG (BF>20; Table S2). These findings indicate viral exchange between HCMC and other provinces both adjacent to and distant from its borders, along with movement of viruses between adjacent provinces outside of HCMC (Figure 2). Significant clustering was also observed in three provinces: DT, TN, and VT, and may explain the overall significance of AI and PS scores detected using BaTS (Table 1).

### Spatial analysis by population density within HCMC and by province

**Figure 3:**
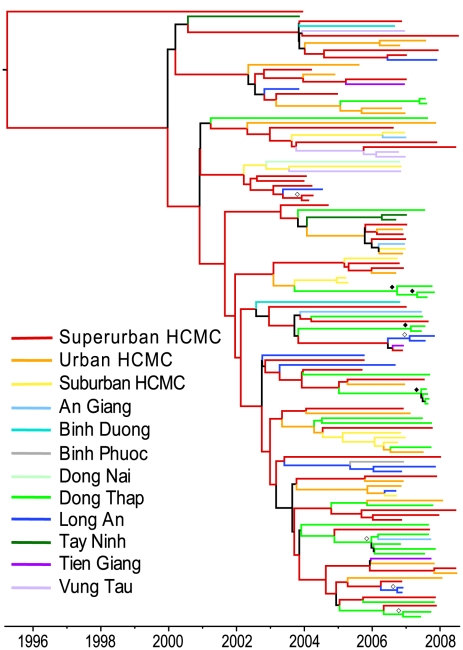
MCC phylogeny of the DENV-2 Asian I genotype in southern Viet Nam (2003–2008) according to population density (within HCMC) or province of sampling. Tips are colored by urban level (within HCMC) or province of sampling. Internal branches are colored based on the reconstructed ancestral state as estimated by the reversible diffusion model. Branches colored black indicate Bayesian posterior probabilities less than 0.85. Estimated support for the reconstructed ancestral state is indicated by open (>95%) and closed (>85%) diamonds.

**Figure 4:**
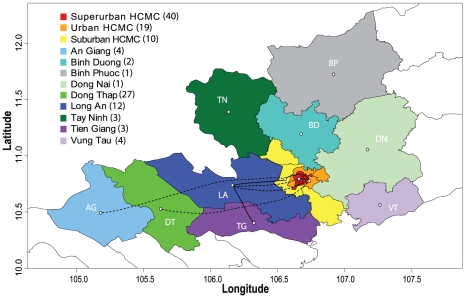
DENV-2 Asian I genotype dispersal across a population density gradient in HCMC and southern Viet Nam. Map showing significant pathways of diffusion estimated between three regions of gradated population density within HCMC and the remaining nine provinces of southern Viet Nam. Solid lines indicate diffusion pathways among regions with a shared border. Dashed lines indicate diffusion pathways among regions that do not have a shared border. Numbers in parentheses indicate the number of sequences included in each group.

When Asian I viruses were categorized according to population density in HCMC and by province elsewhere (three HCMC groups, nine provinces), various patterns emerged. The highly populated area of HCMC showed two strongly supported pathways of diffusion: between the Superurban and Urban districts, and between the Urban and Suburban districts (BF>30). In contrast, viral exchange between Superurban and Suburban HCMC was not supported. Significant diffusion pathways were also observed between regions of HCMC and provinces both distant and adjacent in the Mekong Delta region, while one significant pathway was detected between two adjacent provinces west of HCMC (AG and Suburban HCMC, DT and Urban HCMC, LA and Superurban HCMC, LA and Urban HCMC, LA and Suburban HCMC, and LA and TG, BF>15; Table S3, Figures 3 and 4). These relationships largely corresponded to those detected in the provincial analysis. Sampling bias did not appear to greatly influence these results, or those by province, as similar results were obtained when overrepresented locations were subsampled (Figure S2).

### Spatial analysis by geographic proximity and population density-based district groupings within HCMC and by province

**Figure 5:**
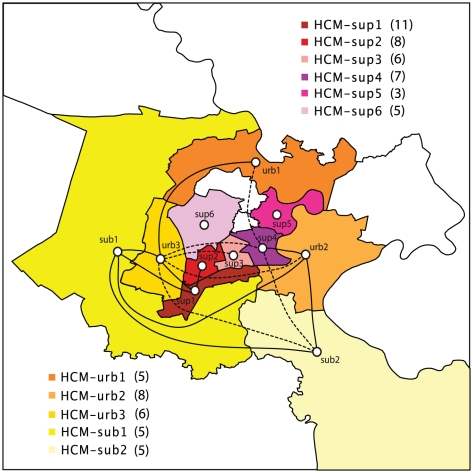
DENV-2 Asian I genotype dispersal among districts of Ho Chi Minh City, Viet Nam. Map indicating significant pathways of diffusion estimated between district groups within HCMC. Solid lines indicate diffusion pathways among areas with a shared border. Dashed lines indicate diffusion pathways among areas that do not have a shared border. Numbers in parentheses indicate the number of sequences included in each group.

**Figure 6:**
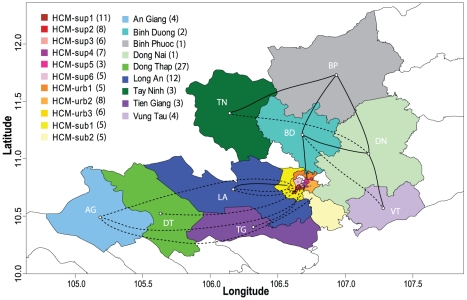
DENV-2 Asian I genotype dispersal among districts of Ho Chi Minh City and provinces of southern Viet Nam. Map showing significant pathways of diffusion estimated between district groups within HCMC and provinces of southern Viet Nam. Solid lines indicate diffusion pathways among regions with a shared border. Dashed lines indicate diffusion pathways among regions that do not have a shared border. Numbers in parentheses indicate the number of sequences included in each group.

Use of finer-scale spatial classification within HCMC (11 geography- and population-based district groupings, nine provinces) revealed that samples from most geographic locations (both within and outside of HCMC) were dispersed throughout the phylogeny of the Asian I genotype (Figure S3). Within HCMC, significant diffusion pathways were detected across the city (BF>15, Table S4), with strong mixing between areas of similar population density and relatively few diffusion pathways connecting districts in the lowest and highest population density categories, and hence suggestive of a gravity diffusion model (Figure 5). Additionally, seven of the eight estimated diffusion pathways with the highest support connect areas with shared borders. With respect to exchange between HCMC and the rest of southern Viet Nam, five significant diffusion pathways between four of the highest density districts of HCMC and four provinces (HCM-sup1 and LA each represented in two pathways) were inferred. Significant diffusion was also detected between three lower-density districts and outer provinces: AG and HCM-urb3, LA and HCM-sub1, and AG and HCM-sub2. Of these, only LA and HCM-sub1 are located directly adjacent to one another. Among the provinces outside of HCMC, significant diffusion pathways were estimated between BD and BP, BD and DN, BD and VT, BP and DN, BP and TN, DN and TN, and DN and VT (Figure 6). No viral migration was identified among these sites in our coarse-scale analyses, and the statistical significance of these inferred diffusion pathways at the finest scale may result from the relatively small numbers of isolates from these areas and potential over-parameterization of the spatial model. Indeed, there is an inherent increase in uncertainty in these models as additional geographic groups are defined. The loss of a significant link between LA and TG in this analysis (and between AG and DT in the previous analysis) also likely reflects the increased complexity of the phylogeographic model following the addition of spatial groups; relatively low, but significant, Bayes factors were calculated for links between these provinces in the provincial analysis (Table S1).

Importantly, the general effects of the number and scale of spatial parameters on phylogeographic model inference are currently under investigation, such that the results of this finest-scale analysis, particularly those that are inconsistent with earlier analyses, should be considered as provisional. It is also possible that these changes reflect general trends in mixing between HCMC and other communities within the region that become clearer as population characteristics of the large urban area of HCMC are taken into account in the analysis. These uncertainties not withstanding, it is interesting to note that the new connections observed at this finest spatial level are compatible with a previously undetected transmission network among the provinces east and north of HCMC, largely industrial areas that are geographically isolated from the Mekong Delta provinces showing consistent viral exchange with HCMC.

### Distance and gravity model-based spatial analysis

Finally, although some movement patterns are clearly suggestive of gravity-like dynamics, and particularly within HCMC (see above), distance and gravity model-informed priors did not result in an overall better fit to the data than the default constant rate priors (as reflected in marginal log likelihoods, Table S5). Notably, gravity model-informed priors were statistically superior to distance-informed priors. This result is not entirely surprising, as physical distances alone may poorly reflect the complexity of human population movements [38]. Additionally, both our distance and gravity model calculations were based on official government boundaries and population estimates for relatively large geographic areas, but samples (and populations) were not uniformly distributed across each of these locations, and the use of distances between centroids may not correspond to actual distances between populated areas using roads or waterways, although we would expect these to be similar. Importantly, similar results were obtained when distances were calculated between the centroids of our viral populations instead of provinces, with slightly increased and slightly decreased likelihoods apparent when gravity model-informed and distance-informed priors were used, respectively (data not shown).

## Discussion

This study documents aspects of the diffusion of DENV-2 throughout a large, highly endemic region of southern Viet Nam, and provides insight into the capacity of genome sequence data to capture potentially important trends in the spatial and temporal dynamics of DENV. Our phylogeographic analysis suggests that the Asian I genotype of DENV-2, which likely entered southern Viet Nam in the late 1990s from elsewhere in Southeast Asia and recently displaced the American/Asian genotype as the predominant DENV-2 lineage in the region [15], has circulated consistently in the high population density region of HCMC since at least 2000 and rapidly spread to populations across the region during the process of lineage replacement. During the period of sampling, specifically from 2003–2007, dengue incidence nearly doubled across southern Viet Nam; this increase was mostly associated with DENV-2 infection. Data suggest a high force of infection attributable to the Asian I lineage during most of this period, which corresponds with the displacement of the American/Asian genotype [15]. The timeframe and spatial scale at which this clade replacement event was observed indicate that the Asian I lineage spread relatively rapidly into populations across the region following its introduction, and suggest that human movement likely played a significant role in the dispersal of the novel lineage. A similar process of genotype replacement appears to have occurred on different timescales in both Thailand and Cambodia, either of which may act as a source population for southern Viet Nam [15]. Unfortunately, a detailed spatial analysis of the American/Asian genotype was not possible due to the smaller number of samples isolated during the study period.

Major urban areas of Southeast Asia have previously been proposed to play central roles in DENV epidemics, harboring the greatest viral genetic diversity and population sizes sufficiently large to allow sustained outbreaks that may subsequently spread to more rural areas, and potentially acting as harbingers of epidemic dengue activity in a given season [39], [40]. While the reversible nature of the diffusion pathways estimated in this study does not allow the directionality of viral movement to be determined, our results are clearly compatible with the idea that HCMC acts as a driver of viral diffusion into other locales in southern Viet Nam, either as a major source population for dengue viruses or as a mixing ground into which viruses are trafficked by the movement of migrants and visitors from rural areas into the city and are subsequently relayed out to other parts of the country through mosquito and human movements. Our analysis suggests the former of these to be more likely, as location reconstruction showed strong support for the deepest branches of the MCC tree originating in HCMC. The very large population (∼6.6 million; 3155 persons/km^2^) [24] of this urban area likely contains sufficiently high numbers of susceptible hosts to allow sustained year-round hyperendemic transmission, thereby providing ample opportunities for DENV to evolve within the city and its surrounding suburban districts. Additionally, the greater connectivity between HCMC and the rest of Southeast Asia, manifest in such features as the number of airline routes, will obviously facilitate the importation of new viral lineages into this population. Finally, our study indicates that HCMC may consistently maintain multiple viral lineages of a single DENV genotype throughout the year. Indeed, it is striking that our BaTS analysis detected no large monophyletic groups (i.e. >4.46 sequences in the provincial analysis) within HCMC at any spatial scale; lineages in which HCMC was dominant and fell basal on the tree often included at least one virus obtained from a resident of another province, providing additional evidence for diffusion out of HCMC.

Notably, its role as the center of commerce and industry in the region makes HCMC a major acceptor of human in-migration from more rural areas, particularly to districts labeled ‘urban’ and ‘suburban’ in this analysis [41], and occasional movements of these migrants between HCMC and their home provinces may in part fuel rapid dispersal of DENV to distant provinces such as An Giang and Dong Thap, as well as to northern portions of the country. Our analysis may to some extent reflect this movement, as significant diffusion pathways were consistently detected between these provinces and urban and suburban HCMC. It is also possible that these movements introduce new lineages from rural into urban areas, as has been suggested for the malaria parasite [42], thus allowing them to become established within large populations and potentially be exported to new communities across the region. Unfortunately, the relatively small numbers of sequences from many of the communities outside of HCMC preclude us from determining whether this movement from rural to urban areas plays a major role in the spatial dynamics of DENV transmission. Further, although the substantial timescales of some of these long distance migration events mean that we are unable to reject the possibility that numerous small-scale mosquito-based transmission cycles resulted in virus dispersal over long distances, the lack of closely related isolates from intermediate geographic areas suggests that the viral lineages may have traveled across these provinces rapidly enough that transmission chains were not established within the population, as would occur with human rather than mosquito movement. Greater numbers of samples from communities outside of HCMC and mosquito populations from across the region would allow us to explore this further, and to potentially confirm the presence or lack of specific viral lineages in intermediate areas. While the Asian I lineage appears to have become established within the population, ongoing sampling in southern Viet Nam would clearly allow us to capture the introduction and dispersal events of future DENV lineages, potentially on a finer spatial and temporal scale than possible here.

Despite the relatively small sample size, phylogenetic clustering of viruses, itself an indicator of potential *in situ* evolution, was clearly detected among viruses isolated from residents of Dong Thap, Tay Ninh, and Vung Tau. Dong Thap province in particular yielded several small monophyletic groups, as well as one well-supported clade that contained a sequence from An Giang, its neighbor to the west. This suggests that locales that are relatively geographically isolated from highly populated urban areas may experience some population subdivision similar to that observed at a smaller spatial scale in northern Thailand [12]. The existence of a viral clade isolated exclusively from this region in late 2006 and late 2007 with an estimated divergence time of approximately two years prior to isolation (data not shown; TMRCA 95%HPD from 2005.2 to 2006.1) suggests that local transmission networks in semi-rural areas such as these western provinces may be capable of maintaining virus populations and fueling DENV evolution over multiple seasons. In addition, the detection of multiple DENV-2 lineages in Dong Thap and Long An extending through 2006 and 2007, some of which appear to have local histories dating back to previous dengue seasons, indicates that several introductions of Asian I DENV-2 have likely occurred in these provinces in recent years. Similar findings were reported from a mixed urban-rural environment in Thailand [12]. More generally, these findings suggest that Dong Thap and rural regions of Southeast Asia may act as sink populations, dependent upon local seropositivity rates at a given time, with HCMC and other major urban city centers functioning as DENV source populations for surrounding areas.

Within HCMC, the patterns of DENV movement between the three regions of varying population density are consistent with a gravity model of virus dispersal, with viruses moving down (or up) gradated population density categories even though all three regions share borders. This trend of movement across a gradient of population densities is largely upheld in the finer-scale analysis within the city, with 13 of 15 significant viral diffusion pathways in the city detected between areas of similar population density or one degree removed, and the majority of viral movement occurring between adjacent districts. Using a similar phylogeographic approach, Balmaseda et al. also observed viral movement between adjacent neighborhoods in a cohort study in Managua, Nicaragua, with some exchange occurring between more distant neighborhoods, likely attributable to transportation networks and migrant workers moving within the community [13]. Similar to numerous other genetic and epidemiological studies [12], [14], [43], [44], the analysis in Nicaragua revealed marked spatial clustering in relatively small areas (in this case, by neighborhood). Although the sampling regime undertaken here does not allow us to fully capture short transmission networks, our observation of virus dispersal over relatively short distances and between adjacent districts within HCMC highlights probable roles for local mosquito populations and small-scale human movements in the diffusion of DENV in this highly urban area.

In sum, our study indicates that DENV moves relatively freely among human populations in southern Viet Nam, over both long and short distances, and hence suggests a major role for anthropogenic factors and urban areas as drivers of DENV dispersal in Southeast Asia. However, the relative isolation of some areas directly adjacent to well-connected areas is not well understood, such that it is difficult to make strong conclusions on the predictability of DENV transmission dynamics within this highly endemic region; spatio-temporal variation in seropositivity may play a significant role in preventing new viral lineages from being established in some populations, and the contribution of this factor should be investigated further in future studies. Importantly, neither distance-based nor gravity models were able to explain the full complexity of the transmission dynamics, although a gravity model showed a slightly better fit to our data than did the distance-based models for both the provincial and population density-based analyses. The improved fit of the model upon the integration of population data provides further evidence that human population movement is an important factor acting on DENV dispersal in the region. Thus, it is possible that increased information on short- and long-term human population movements between rural and urban areas may provide a model with improved predictive power for estimating the future spatial spread of DENV over this area. The use of transportation information, including distances by land (such as road distance) and water travel, as well as relative costs of travel between these areas, may also increase the power of these models to determine important viral migration pathways. In the absence of this information, DENV transmission dynamics within and across cities and rural areas are difficult to predict, as many factors, including human population densities and movement, population immunity, mosquito densities and dispersal, and the seasonality of dengue transmission intensity, as well as other unknown factors, may affect the rates of virus dispersal and establishment of new transmission networks in a locality. As these data represent the initial results of an ongoing study, the isolation of additional DENV sequences over the coming years will allow us to investigate the spatial relationships among viral lineages circulating within the country in greater detail, and may enable us to determine how specific population movement patterns such as seasonal migration and international travel affect the dispersal of viral lineages throughout the region.

## Supporting Information

Figure S1 Genetic and geographic distances among complete coding region sequences of DENV-2. Each pair is represented as a point, with the color of the point indicating the year of sampling. (a) Asian I DENV-2. Colors represent: 2003, black; 2004, blue; 2005, cyan; 2006, green; 2007, yellow, 2008, red; all years, grey. (b) American/Asian DENV-2. Colors represent: 2001, black; 2002, blue; 2003, cyan; 2004, green; 2005, yellow, 2006, red; all years, grey.
(3.48 MB EPS)

Figure S2 Results of the phylogeographic analysis on subsampled populations of DENV-2, Asian I genotype. (a) Phylogeographic analysis by province. (b) Phylogeographic analysis by urban levels within HCMC and by province outside of HCMC.
(0.97 MB EPS)

Figure S3 MCC phylogeny of the Asian I genotype in southern Viet Nam (2003–2008) according to district group (within HCMC) or province of sampling. Tips are colored by district group (within HCMC) or province of sampling. Internal branches are colored based on the reconstructed ancestral state as estimated by the reversible diffusion model. Branches colored black indicate Bayesian posterior probabilities less than 0.85. Estimated support for the reconstructed ancestral state is indicated by open (>95%) and closed (>85%) diamonds.
(0.48 MB EPS)

Figure S4 MCC phylogeny of the American/Asian genotype in southern Viet Nam (2001–2006) according to province of sampling. Tips are colored by province of sampling. Internal branches are colored based on the reconstructed ancestral state as estimated by the reversible diffusion model. Branches colored black indicate Bayesian posterior probabilities less than 0.85. Estimated support for the reconstructed ancestral state is indicated by open (>95%) and closed (>85%) diamonds.
(0.31 MB EPS)

Figure S5 MCC phylogeny of the American/Asian genotype in southern Viet Nam (2001–2006) according to population density (within HCMC) or province of sampling. Tips are colored by urban level (within HCMC) or province of sampling. Internal branches are colored based on the reconstructed ancestral state as estimated by the reversible diffusion model. Branches colored black indicate Bayesian posterior probabilities less than 0.85. Estimated support for the reconstructed ancestral state is indicated by open (>95%) and closed (>85%) diamonds.
(0.36 MB EPS)

Table S1 GenBank accession numbers, year, and province of sampling of DENV-2 genome sequences used in this study.
(0.19 MB DOC)

Table S2 Results of geographic diffusion model for DENV-2, Asian I genotype; locations given by province.
(0.05 MB DOC)

Table S3 Results of geographic diffusion model for DENV-2, Asian I genotype; locations given by urban level within HCMC or province.
(0.06 MB DOC)

Table S4 Results of spatial diffusion model for DENV-2, Asian I genotype; locations given by district group within HCMC or province.
(0.10 MB DOC)

Table S5 Model exploration for the Asian I genotype at two geographic levels.
(0.04 MB DOC)
