# Supplementary material for: Phylogeography of Recently Emerged DENV-2 in Southern Viet Nam
Source: PLoS Negl Trop Dis. 2010 Jul 27;4(7):e766. doi: 10.1371/journal.pntd.0000766 (PMC2910671; doi:10.1371/journal.pntd.0000766)
Supplement: Table S1 — GenBank accession numbers, year, and province of sampling of DENV-2 genome sequences used in this study. (0.19 MB DOC) [file pntd.0000766.s006.doc]

**Table S1. GenBank accession numbers, year, and province of sampling of DENV-2 genome sequences used in this study.**

| **GenBank Accession** | **Sequence ID** | **Year of sampling** | **Province** |
| --- | --- | --- | --- |
| EU482445 | V1000 | 2006 | HCMC |
| EU482446 | V1001 | 2006 | HCMC |
| EU482447 | V1002 | 2006 | An Giang |
| EU482448 | V1003 | 2006 | HCMC |
| EU482449 | V1004 | 2006 | Dong Nai |
| EU482450 | V1005 | 2006 | Long An |
| EU482451 | V1007 | 2006 | An Giang |
| EU482463 | V917 | 2006 | HCMC |
| EU482464 | V918 | 2006 | Nghe An |
| EU482465 | V919 | 2006 | HCMC |
| EU482466 | V920 | 2006 | HCMC |
| EU482467 | V921 | 2006 | HCMC |
| EU482469 | V924 | 2006 | Vung Tau |
| EU482470 | V925 | 2006 | HCMC |
| EU482471 | V926 | 2006 | HCMC |
| EU482472 | V927 | 2004 | HCMC |
| EU482473 | V928 | 2006 | Dong Thap |
| EU482474 | V929 | 2007 | Dong Thap |
| EU482475 | V930 | 2007 | Dong Thap |
| EU482541 | V997 | 2006 | HCMC |
| EU482542 | V998 | 2006 | HCMC |
| EU482543 | V999 | 2006 | Long An |
| EU482640 | V704 | 2006 | HCMC |
| EU482641 | V704 | 2006 | Binh Duong |
| EU482642 | V705 | 2006 | Long An |
| EU482643 | V706 | 2006 | Tay Ninh |
| EU482644 | V707 | 2006 | Long An |
| EU482645 | V708 | 2006 | HCMC |
| EU482646 | V709 | 2006 | HCMC |
| EU482647 | V710 | 2006 | Vung Tau |
| EU482648 | V711 | 2006 | HCMC |
| EU482649 | V712 | 2006 | Vung Tau |
| EU482650 | V713 | 2006 | HCMC |
| EU482650 | V713 | 2006 | Tien Giang |
| EU482651 | V714 | 2006 | Long An |
| EU482652 | V715 | 2006 | HCMC |
| EU482653 | V716 | 2006 | HCMC |
| EU482654 | V717 | 2006 | Binh Phuoc |
| EU482655 | V718 | 2006 | Vung Tau |
| EU482656 | V719 | 2006 | HCMC |
| EU482657 | V720 | 2006 | HCMC |
| EU482658 | V721 | 2006 | Tay Ninh |
| EU482659 | V722 | 2006 | HCMC |
| EU482660 | V723 | 2006 | Tien Giang |
| EU482661 | V724 | 2006 | HCMC |
| EU482662 | V725 | 2006 | HCMC |
| EU482663 | V726 | 2006 | HCMC |
| EU482664 | V727 | 2006 | Dong Nai |
| EU482665 | V728 | 2006 | HCMC |
| EU482666 | V729 | 2006 | Binh Duong |
| EU482667 | V730 | 2006 | HCMC |
| EU482668 | V731 | 2006 | HCMC |
| EU482669 | V732 | 2006 | Dong Nai |
| EU482671 | V734 | 2006 | HCMC |
| EU482672 | V735 | 2006 | HCMC |
| EU482673 | V736 | 2006 | HCMC |
| EU482674 | V737 | 2006 | HCMC |
| EU482675 | V738 | 2006 | HCMC |
| EU482676 | V739 | 2006 | HCMC |
| EU482677 | V740 | 2006 | HCMC |
| EU482678 | V741 | 2006 | HCMC |
| EU482679 | V742 | 2007 | HCMC |
| EU482697 | V771 | 2007 | Dong Thap |
| EU482698 | V772 | 2007 | Dong Thap |
| EU482699 | V773 | 2007 | An Giang |
| EU482700 | V774 | 2007 | Dong Thap |
| EU482701 | V775 | 2007 | Dong Thap |
| EU482702 | V776 | 2007 | Dong Thap |
| EU482703 | V777 | 2007 | Dong Thap |
| EU482704 | V778 | 2007 | Dong Thap |
| EU482705 | V779 | 2007 | Dong Thap |
| EU482774 | V752 | 2004 | HCMC |
| EU482775 | V753 | 2004 | HCMC |
| EU482776 | V754 | 2005 | HCMC |
| EU482777 | V755 | 2005 | HCMC |
| EU482778 | V756 | 2003 | HCMC |
| EU482779 | V757 | 2003 | HCMC |
| EU482780 | V758 | 2003 | HCMC |
| EU482781 | V759 | 2003 | HCMC |
| EU482782 | V760 | 2003 | Long An |
| EU482783 | V761 | 2003 | HCMC |
| EU482784 | V762 | 2003 | HCMC |
| EU482785 | V763 | 2003 | HCMC |
| EU482786 | V764 | 2003 | HCMC |
| EU482787 | V765 | 2003 | HCMC |
| EU482788 | V766 | 2003 | HCMC |
| EU482821 | V827 | 2006 | Binh Duong |
| EU569721 | V1006 | 2006 | HCMC |
| EU660413 | V1507 | 2007 | Dong Thap |
| EU660414 | V1508 | 2007 | Dong Thap |
| EU660415 | V1509 | 2007 | Dong Thap |
| EU660416 | V1511 | 2007 | Dong Thap |
| EU660417 | V1512 | 2007 | Dong Thap |
| EU677137 | V1513 | 2007 | Dong Thap |
| EU677138 | V1514 | 2007 | Dong Thap |
| EU677148 | V1515 | 2007 | Dong Thap |
| EU677149 | V1517 | 2007 | Dong Thap |
| EU687248 | V1518 | 2007 | Dong Thap |
| EU687249 | V1519 | 2007 | Dong Thap |
| EU687250 | V1520 | 2007 | Dong Thap |
| EU726776 | V1522 | 2007 | Dong Thap |
| FJ024452 | V1654 | 2007 | Dong Thap |
| FJ024454 | V1669 | 2007 | Dong Thap |
| FJ024458 | V1674 | 2007 | Dong Thap |
| FJ024461 | V1675 | 2007 | An Giang |
| FJ205877 | V1679 | 2007 | Dong Thap |
| FJ205878 | V1680 | 2007 | Dong Thap |
| FJ205879 | V1682 | 2007 | Dong Thap |
| FJ205880 | V1684 | 2007 | Dong Thap |
| FJ373299 | V1685 | 2007 | Dong Thap |
| FJ390384 | V1690 | 2007 | Dong Thap |
| FJ390385 | V1696 | 2007 | Dong Thap |
| FJ390387 | V1697 | 2007 | Dong Thap |
| FJ410193 | V1699 | 2007 | Dong Thap |
| FJ410195 | V1773 | 2007 | HCMC |
| FJ410200 | V1776 | 2007 | HCMC |
| FJ410202 | V1780 | 2007 | HCMC |
| FJ410208 | V1807 | 2007 | HCMC |
| FJ410215 | V1844 | 2008 | HCMC |
| FJ410217 | V1777 | 2007 | Long An |
| FJ410219 | V1837 | 2007 | HCMC |
| FJ410219 | V1857 | 2007 | HCMC |
| FJ410221 | V1930 | 2007 | HCMC |
| FJ410223 | V1853 | 2007 | Long An |
| FJ410224 | V1858 | 2007 | Long An |
| FJ410228 | V1864 | 2007 | HCMC |
| FJ410233 | V1845 | 2008 | HCMC |
| FJ410237 | V1848 | 2008 | HCMC |
| FJ410241 | V1928 | 2008 | HCMC |
| FJ410259 | V1900 | 2008 | HCMC |
| FJ410288 | V1868 | 2007 | HCMC |
| FJ432726 | V1869 | 2007 | HCMC |
| FJ461305 | V1872 | 2007 | HCMC |
| FJ461309 | V1905 | 2008 | HCMC |
| FJ461311 | V1895 | 2008 | HCMC |
| FJ461314 | V1873 | 2007 | Long An |
| FJ461321 | V1881 | 2007 | HCMC |
| FJ547064 | V1794 | 2007 | HCMC |
| FJ547067 | V1801 | 2007 | Tien Giang |
| FJ562098 | V1818 | 2007 | HCMC |
| FJ859028 | V1796 | 2007 | HCMC |
| FJ873811 | V922 | 2006 | Tien Giang |
| FM210202 | DF768 | 2004 | Long An |
| FM210203 | DF900 | 2003 | HCMC |
| FM210204 | MD903 | 2003 | HCMC |
| FM210205 | MD1533 | 2005 | HCMC |
| FM210206 | MD1600 | 2005 | HCMC |
| FM210207 | MD1619 | 2005 | Long An |
| FM210208 | DF707 | 2003 | HCMC |
| FM210209 | DF726 | 2003 | HCMC |
| FM210210 | DF727 | 2003 | HCMC |
| FM210211 | DF907 | 2003 | Tay Ninh |
| FM210212 | MD902 | 2003 | HCMC |
| FM210213 | MD1504 | 2005 | Tien Giang |
| FM210214 | CSF63 | 2004 | Tien Giang |
| FM210215 | DF380 | 2004 | Long An |
| FM210216 | DF401 | 2004 | HCMC |
| FM210217 | DF404 | 2004 | HCMC |
| FM210218 | DF593 | 2002 | HCMC |
| FM210219 | DF670 | 2003 | HCMC |
| FM210220 | DF674 | 2003 | HCMC |
| FM210221 | DF699 | 2003 | HCMC |
| FM210222 | DF755 | 2004 | Long An |
| FM210223 | DF897 | 2003 | HCMC |
| FM210224 | MD518 | 2001 | HCMC |
| FM210225 | MD594 | 2001 | HCMC |
| FM210226 | MD861 | 2002 | HCMC |
| FM210227 | MD863 | 2002 | Dong Thap |
| FM210228 | MD917 | 2003 | HCMC |
| FM210229 | MD919 | 2003 | HCMC |
| FM210230 | MD944 | 2003 | HCMC |
| FM210231 | MD1240 | 2004 | HCMC |
| FM210232 | MD1270 | 2004 | HCMC |
| FM210233 | MD1272 | 2004 | HCMC |
| FM210234 | MD1366 | 2004 | HCMC |
| FM210235 | MD1515 | 2005 | Tien Giang |
| FM210236 | CSF381 | 2004 | Ben Tre |
| FM210237 | DF657 | 2003 | HCMC |
| FM210238 | MD510 | 2001 | HCMC |
| FM210239 | MD1244 | 2004 | HCMC |
| FM210240 | MD1273 | 2004 | HCMC |
| FM210241 | MD1275 | 2004 | HCMC |
| FM210242 | MD1279 | 2004 | HCMC |
| FM210243 | MD1280 | 2004 | HCMC |
