# Supplementary material for: Phylogeography of Recently Emerged DENV-2 in Southern Viet Nam
Source: PLoS Negl Trop Dis. 2010 Jul 27;4(7):e766. doi: 10.1371/journal.pntd.0000766 (PMC2910671; doi:10.1371/journal.pntd.0000766)
Supplement: Table S2 — Results of geographic diffusion model for DENV-2, Asian I genotype; locations given by province. (0.05 MB DOC) [file pntd.0000766.s007.doc]

**Table S2. Results of geographic diffusion model for DENV-2, Asian I genotype; locations given by province**.

|  | **An Giang** | **Binh Duong** | **Binh Phuoc** | **Dong Nai** | **Dong Thap** | **HCMC** | **Long An** | **Tay Ninh** | **Tien Giang** | **Vung Tau** |
| --- | --- | --- | --- | --- | --- | --- | --- | --- | --- | --- |
| **An Giang** | **-** | NS | NS | NS | 21.5 | NS | NS | NS | NS | NS |
| **Binh Duong** |  | **-** | NS | NS | NS | NS | NS | NS | NS | NS |
| **Binh Phuoc** |  |  | **-** | NS | NS | NS | NS | NS | NS | NS |
| **Dong Nai** |  |  |  | **-** | NS | NS | NS | NS | NS | NS |
| **Dong Thap** |  |  |  |  | - | 3303.7 | NS | NS | NS | NS |
| **HCMC** |  |  |  |  |  | - | 563.6 | NS | NS | NS |
| **Long An** |  |  |  |  |  |  | - | NS | 28.6 | NS |
| **Tay Ninh** |  |  |  |  |  |  |  | - | NS | NS |
| **Tien Giang** |  |  |  |  |  |  |  |  | - | NS |
| **Vung Tau** |  |  |  |  |  |  |  |  |  | - |
| Bayes Factors >15 are indicated. NS denotes a non-significant Bayes Factor (BF<15). | | | | | | | | | | |
