# Supplementary material for: Phylogeography of Recently Emerged DENV-2 in Southern Viet Nam
Source: PLoS Negl Trop Dis. 2010 Jul 27;4(7):e766. doi: 10.1371/journal.pntd.0000766 (PMC2910671; doi:10.1371/journal.pntd.0000766)
Supplement: Table S4 — Results of spatial diffusion model for DENV-2, Asian I genotype; locations given by district group within HCMC or province. (0.10 MB DOC) [file pntd.0000766.s009.doc]

**Table S4. Results of spatial diffusion model for DENV-2, Asian I genotype; locations given by district group within HCMC or province.**

|  | **HCM-sup1** | **HCM-sup2** | **HCM-sup3** | **HCM-sup4** | **HCM-sup5** | **HCM-sup6** | **HCM-urb1** | **HCM-urb2** | **HCM-urb3** | **HCM-sub1** | **HCM-sub2** | **An Giang** | **Binh Duong** | **Binh Phuoc** | **Dong Nai** | **Dong Thap** | **Long An** | **Tay Ninh** | **Tien Giang** | **Vung Tau** |
| --- | --- | --- | --- | --- | --- | --- | --- | --- | --- | --- | --- | --- | --- | --- | --- | --- | --- | --- | --- | --- |
| **HCM-sup1** | - | 32.4 | NS | NS | NS | NS | NS | NS | 19.3 | 19.2 | NS | NS | NS | NS | NS | 86.4 | 31.0 | NS | NS | NS |
| **HCM-sup2** |  | - | NS | NS | NS | NS | NS | NS | NS | NS | NS | NS | NS | NS | NS | NS | 48.2 | NS | NS | NS |
| **HCM-sup3** |  |  | - | NS | NS | NS | NS | NS | 18.3 | NS | NS | NS | NS | NS | NS | NS | NS | NS | 46.6 | NS |
| **HCM-sup4** |  |  |  | - | NS | NS | 23.1 | 18.7 | 17.8 | NS | 17.4 | 18.6 | NS | NS | NS | NS | NS | NS | NS | NS |
| **HCM-sup5** |  |  |  |  | - | NS | NS | NS | NS | NS | NS | NS | 15.4 | NS | NS | NS | NS | NS | NS | NS |
| **HCM-sup6** |  |  |  |  |  | - | NS | NS | NS | NS | NS | NS | NS | NS | NS | NS | NS | NS | NS | NS |
| **HCM-urb1** |  |  |  |  |  |  | - | NS | 15.3 | NS | NS | NS | NS | NS | NS | NS | NS | NS | NS | NS |
| **HCM-urb2** |  |  |  |  |  |  |  | - | 15.4 | 28.9 | 18.4 | NS | NS | NS | NS | NS | NS | NS | NS | NS |
| **HCM-urb3** |  |  |  |  |  |  |  |  | - | 15.2 | 16.9 | 17.8 | NS | NS | NS | NS | NS | NS | NS | NS |
| **HCM-sub1** |  |  |  |  |  |  |  |  |  | - | 22.1 | NS | NS | NS | NS | NS | 18.2 | NS | NS | NS |
| **HCM-sub2** |  |  |  |  |  |  |  |  |  |  | - | 27.4 | NS | NS | NS | NS | NS | NS | NS | NS |
| **An Giang** |  |  |  |  |  |  |  |  |  |  |  | - | NS | NS | NS | NS | NS | NS | NS | NS |
| **Binh Duong** |  |  |  |  |  |  |  |  |  |  |  |  | - | 16.4 | NS | NS | NS | NS | NS | 15.8 |
| **Binh Phuoc** |  |  |  |  |  |  |  |  |  |  |  |  |  | - | 21.3 | NS | NS | 15.2 | NS | NS |
| **Dong Nai** |  |  |  |  |  |  |  |  |  |  |  |  |  |  | - | NS | NS | 17.7 | NS | 15.2 |
| **Dong Thap** |  |  |  |  |  |  |  |  |  |  |  |  |  |  |  | - | NS | NS | NS | NS |
| **Long An** |  |  |  |  |  |  |  |  |  |  |  |  |  |  |  |  | - | NS | NS | NS |
| **Tay Ninh** |  |  |  |  |  |  |  |  |  |  |  |  |  |  |  |  |  | - | NS | NS |
| **Tien Giang** |  |  |  |  |  |  |  |  |  |  |  |  |  |  |  |  |  |  | - | NS |
| **Vung Tau** |  |  |  |  |  |  |  |  |  |  |  |  |  |  |  |  |  |  |  | - |
| Bayes Factors >15 are indicated. NS denotes a non-significant Bayes Factor (BF<15). | | | | | | | | | | | | | | | | | | | | |
