# Supplementary material for: Phylogeography of Recently Emerged DENV-2 in Southern Viet Nam
Source: PLoS Negl Trop Dis. 2010 Jul 27;4(7):e766. doi: 10.1371/journal.pntd.0000766 (PMC2910671; doi:10.1371/journal.pntd.0000766)
Supplement: Table S5 — Model exploration for the Asian I genotype at two geographic levels. (0.04 MB DOC) [file pntd.0000766.s010.doc]

**Table S5. Model exploration for the Asian I genotype at two geographic levels.**

| **Model** | **Provinces** | **Urban level within HCMC & Provinces** |
| --- | --- | --- |
|  | **ML (stdev)** | **ML (stdev)** |
| **Constant, BSSVS** | **-31546.388 (0.286)** | **-31614.603 (0.218)** |
| Distance-informed, Fixed | -31563.203 (0.292) | -31633.089 (0.318) |
| Distance-informed, MGP | -31560.144 (0.296) | -31633.507 (0.278) |
| Gravity model-informed, Fixed | -31565.760 (0.306) | -31629.136 (0.367) |
| Gravity model-informed, MGP | -31559.071 (0.251) | -31639.873 (0.285) |
| Values indicate log marginal likelihoods (ML) and standard deviations, with the best supported model shown in bold. | | |
